# A retrospective medical chart review of clinical outcomes in children and adolescents with attention-deficit/hyperactivity disorder treated with guanfacine extended-release in routine Canadian clinical practice

**DOI:** 10.1186/s13034-021-00402-5

**Published:** 2021-10-04

**Authors:** Judy van Stralen, Simerpal K. Gill, Christopher J. Reaume, Kenneth Handelman

**Affiliations:** 1 Center for Pediatric Excellence, 206-1637 Woodroffe Avenue, Ottawa, ON K2G 1W2 Canada; 2 Takeda Canada Inc., Toronto, ON Canada; 3 Shire Pharma Canada ULC, A Takeda Company (Now Takeda Canada Inc.), Toronto, ON Canada; 4 Halton Healthcare, Oakville, ON Canada

**Keywords:** ADHD, ADHD symptoms, Guanfacine extended-release, Non-stimulant, Chart review

## Abstract

**Objective:**

This study evaluated clinical outcomes in children and adolescents with attention-deficit/hyperactivity disorder (ADHD) treated with the α_2_-adrenoceptor agonist guanfacine extended-release (GXR) in routine Canadian clinical practice.

**Methods:**

This retrospective chart review focused on patients with ADHD aged 6–17 years initiating treatment with GXR as monotherapy or adjunctive therapy. Patients were followed for up to 12 months after GXR initiation and, if they had received prior ADHD pharmacotherapy, for 12 months before GXR initiation. The primary outcome was change in ADHD symptoms and functionality based on physician assessments, classified as improvement, no change, or worsening relative to the time of GXR initiation. Treatment-emergent adverse events (TEAEs) were evaluated. Clinical outcomes were also analyzed post hoc according to whether GXR treatment was received as monotherapy or adjunctive therapy, and by select psychiatric comorbidities. Exploratory analyses were conducted in patients who had received prior ADHD pharmacotherapy to evaluate clinical outcomes after initiating GXR.

**Results:**

Improvements in ADHD symptoms were reported for 232/330 (70.3%) patients. Functional improvements in school performance and home life were reported for 213/330 (64.5%) and 209/330 (63.3%) patients, respectively. The most frequent TEAEs (≥ 5%) were somnolence, headache, insomnia, presyncope, and decreased appetite. Improvements in ADHD symptoms were observed when GXR was received as either monotherapy (35/60 [58.3%]) or adjunctive therapy (197/270 [73.0%]). Improvements in ADHD symptoms and functionality were observed in the majority of patients with select psychiatric comorbidities. Among patients who had experienced worsening of symptoms with prior ADHD pharmacotherapy, 44/54 (81.5%) experienced symptom improvement, 33/44 (75.0%) who had previously experienced worsening of school performance improved, and 34/48 (70.8%) who had previously experienced worsening of home life improved.

**Conclusion:**

In Canadian routine clinical practice, most children and adolescents with ADHD treated with GXR experienced improvements in ADHD symptoms and in functionality both at school and at home.

**Supplementary Information:**

The online version contains supplementary material available at 10.1186/s13034-021-00402-5.

## Introduction

Attention-deficit/hyperactivity disorder (ADHD) is a neurodevelopmental disorder with childhood onset characterized by a pervasive pattern of age-inappropriate and excessive inattention and hyperactivity/impulsivity [1]. The estimated worldwide prevalence of ADHD is ~ 5.3% [2]. From 2003 to 2011 ADHD incidence grew an average of 5% yearly, due to increased recognition and diagnosis [3]. Canadian ADHD prevalence rates are consistent with worldwide estimates; a recent retrospective review of medical records from Ontario found a prevalence rate of 5.4% (males, 7.9%; females, 2.7%) [4].

The socioeconomic burden of ADHD is considerable, impairing many aspects of children’s lives and their families, and negatively affecting academic performance and interpersonal relationships. Specific manifestations may include inattention and distractibility in daily routines, impulsive behavior, or missing social cues [5]. In young people and adults, ADHD may also be associated with higher risk of self-harm, traffic accidents, delinquency, and substance misuse [6].

In the United States, healthcare costs for children and adolescents with ADHD have been estimated at $US 7.9 billion annually (2005 costs) [7]. Additionally, total costs, including excess costs associated with healthcare and work loss among family members of people with ADHD have been estimated to be $US 34.6 billion annually (2000 costs) [8]. In Canada, total ADHD-associated costs are estimated to exceed $CA 7 billion [9].

Both psychosocial and pharmacologic interventions are used for ADHD treatment. Combination therapy with approved medication and behavioral therapy is the preferred model for children aged ≥ 6 years and adolescents [10]. Stimulants such as methylphenidate and amphetamines are the mainstay of ADHD therapy. However, a single stimulant may not adequately control ADHD symptoms in 25–30% of children [11, 12]. Impairments associated with residual ADHD symptoms in patients suboptimally treated with stimulants, and the need to optimize therapy, are becoming increasingly recognized [13]. Furthermore, some patients may not tolerate stimulants. In Canada, non-stimulant alternatives such as atomoxetine and guanfacine extended-release (GXR) are available as monotherapy for patients who do not respond to or cannot tolerate first-line stimulant therapy [14]. As a selective α_2_-adrenoceptor agonist, GXR can also be used as adjunctive therapy to stimulants [14, 15].

Real-world evidence provides valuable knowledge beyond the ideal conditions of a clinical trial, and several such studies have assessed usage patterns and costs of non-stimulants in patients with ADHD [16, 17]. However, GXR utilization in routine clinical care is less clear. Real-world experience with GXR has accumulated in Canada, where it has been available to treat ADHD since 2013 as an extended-release tablet and is approved for patients aged 6–17 years for the treatment of ADHD either as monotherapy or adjunctive therapy to stimulants if response to stimulants is suboptimal [15]. In randomized controlled clinical trials, GXR has been effective and well tolerated as monotherapy and adjunctive therapy [18–25]. Additionally, GXR lacks potential for abuse and may be used with stimulant medications [26]. The purpose of this study was to evaluate clinical outcomes in children and adolescents with ADHD treated with the α_2_-adrenoceptor agonist GXR in routine Canadian clinical practice.

## Methods

### Study design and population

This study was a retrospective medical chart review in children and adolescents with ADHD recruited from 10 sites by Canadian community- or hospital-based pediatricians, child psychiatrists, or family doctors specializing in ADHD. Included children (6–12 years) and adolescents (13–17 years) had diagnosed ADHD, had initiated treatment with GXR (monotherapy or adjunctive therapy), and had ≥ 6 months of medical chart data post-initiation of GXR (with ≥ 1 follow-up; if applicable, this included the visit when GXR was discontinued). Patients could have been treatment naive or received other pharmacotherapy before GXR initiation. In treatment-naive patients, the observation period of the chart review began at treatment initiation, whereas in patients who had received prior ADHD pharmacotherapy, the observation period started 12 months prior to GXR initiation. The chart review observation period ended at ≤ 12 months after GXR initiation. If GXR was discontinued, the reason for discontinuation was noted but no other follow-up data were extracted after the discontinuation visit.

### Data collection and outcome measures extracted

Data were transcribed from patient charts to case report forms (CRFs) by the site investigator or designated staff and sent to a central database. No patient identifying information was recorded; patients were given encrypted random study identification numbers. Data on patient demographics and clinical characteristics were extracted from patient charts per standard of care for ADHD. At ADHD diagnosis, these data included diagnosis date, sociodemographics (age, gender, ethnicity, location of residence [urban vs rural]), medical history, and comorbidities. At GXR initiation, data extracted included reason for GXR initiation, prior non-pharmacologic treatments for ADHD or comorbid conditions, and prior pharmacologic treatments, including stimulants, non-stimulants, and atypical antipsychotics (AAPs) for ADHD or comorbid psychiatric conditions/symptoms. At GXR initiation and for its duration, covariates included GXR information (dose, frequency, dose/frequency changes and reasons, termination date and reason) and pharmacologic treatment for ADHD or comorbid psychiatric symptoms. If GXR was discontinued, reasons for discontinuation and any subsequent ADHD-related pharmacologic treatment regimen were recorded, and no additional follow-up details were extracted following the discontinuation visit.

Extracted outcome data were from the time prior to (for patients receiving prior pharmacologic treatment only), at GXR initiation, and for its duration (≤ 12 months). ADHD symptoms and functionality were measured using the General Physician Symptom Assessment and General Physician Functional Assessment (school grades/performance and homelife), respectively. Rating scale data on ADHD symptoms and functionality, if available in patient charts, were also extracted and evaluated. Symptom ratings scales included Swanson, Nolan, and Pelham-IV (SNAP-IV-26) [27], National Institute for Children’s Health Quality (NICHQ) Vanderbilt Assessment Scale and Follow-up-Parent Information [28], and Conners 3-Parents (Conners 3-P) [29]. The Weiss Functional Impairment Rating Scale-Parent Report (WFIRS-P) was used to measure functionality [30]. To minimize recall bias related to subjective assessment of symptomatology (as extracted from patients’ charts in the absence of validated scales), outcome recorders were requested to categorize consistently the patient’s response, as indicated in the patient charts. Outcome recorders had prior training including how to record consistently a category for patient responses based on data in patient charts. ADHD symptoms and functionality were categorized as “improvement”, “no change”, or “worsening” based on rating scales in medical charts, and/or clinician’s notes. An improvement/worsening of symptoms or functionality for two consecutive visits was categorized as “improved” or “worsening”, respectively.

Treatment-emergent adverse events (TEAEs) occurring during GXR treatment were coded using the Medical Dictionary for Regulatory Activities (MedDRA) version 20 and described by the proportion of patients experiencing a TEAE within each Preferred Term and System Organ Class.

### Sample size

Charts from a minimum of 252 and maximum of 444 patients were required. In research by Cutler et al. [31], symptomatic remission was achieved in 62.2% versus 46.1% of patients with ADHD aged 6–17 years with suboptimal response to stimulants treated with GXR plus stimulant versus placebo plus stimulant, respectively. Based on these data, the assumption was that ~ 60% of patients in this study would experience symptom improvement. To detect a significant difference in response with α = 5% and 80% power, 202 evaluable patients were required. Allowing that required data would not be available in 25% of charts, data from a minimum of 252 charts were required.

In Cutler et al. [31], 77.3% of patients experienced a TEAE. Assuming a similar rate in our study with 252 patients, the precision of estimate as assessed with the width of the 95% confidence interval (CI) would be 13.5% of the estimate (0.104), which is within acceptable limits. To achieve 10% precision (0.078), 444 patients would be required. With 252 and 444 patients, the probability of detecting a TEAE with true incidence of 1% was 92% and 99%, respectively.

### Statistical methods

Summary statistics were calculated for all study variables, including mean, median, standard deviation (SD), 95% CI of the mean, and frequency distributions for continuous scale variables.

The primary analysis was change in ADHD symptoms and functionality, assessed by the proportion of patients showing improvement versus no change versus worsening. For patients without previous ADHD treatment, this categorization was based on assessment of the patient compared with the previous visit (beginning with the comparison of the first assessment post-GXR initiation to status at initiation). For patients who had received prior ADHD pharmacologic treatment, the pre-GXR value corresponded to the overall response (improved, no change, or worse) to the last treatment regimen administered prior to GXR initiation. An improvement/worsening of symptoms or function was defined as a categorization of patient response as improved or worse, respectively, for two consecutive visits. The best symptom and functionality responses were described, corresponding to the best respective responses registered during the post-GXR initiation observation period while receiving GXR.

Post-hoc analyses (unspecified in the protocol) included assessments of symptoms and functionality whether GXR treatment was monotherapy/adjunctive therapy, if patients had been initiated on GXR to reduce use of AAPs, and according to psychiatric comorbidities of interest (oppositional defiant disorder, learning disability, anxiety, and autism spectrum disorder).

Data from patients who had received ADHD pharmacologic treatment before GXR and had ≤ 12 months of data following GXR initiation, a subset not considered in power calculations, were examined in exploratory analyses. The objective was to assess the impact on clinical outcomes of switching to GXR or adding GXR to current ADHD treatment. The pre-GXR value (prior period) corresponded to the overall response (improved, no change, or worse) to the last treatment regimen administered before GXR initiation. The analyses detailed for the primary objective were repeated in this subgroup, although the post-hoc breakdown of results (by monotherapy/adjunctive therapy, etc.) was not conducted. The comparison of the response rate (improvement vs no change vs worsening) of General Physician Symptom and Functional Assessments before and after GXR initiation was conducted with the extension of McNemar’s test (Bowker’s test); two-tailed test. The null hypothesis was that the percentages are equal before and after GXR initiation, i.e., during the baseline period and post-GXR initiation observation period (exploratory analysis).

As this was a retrospective chart review, no missing data could be retrieved. However, cross-validation was performed, and erroneous data were clarified with physicians. Partial dates were completed using the most conservative approach when required to determine duration of observation, treatment exposure, and incidence of adverse events. Otherwise, all analyses were conducted on available data with no data imputation.

## Results

Study sites were activated on a rolling basis beginning on 5 October 2016; database lock occurred on 16 June 2017.

### Patient characteristics

In total, 330 charts were screened, and all patients were included in the analysis (Additional file 1: Table S1). Approximately 92% and 40% had prior pharmacologic and non-pharmacologic ADHD treatment within the 12 months preceding GXR initiation, respectively. The majority of patients were male Caucasian children (6–12 years) living in Ontario.

Comorbid psychiatric conditions were reported for 215/330 (65.2%) patients. The most frequently reported conditions experienced by ≥ 5% of patients were oppositional defiant disorder (n = 92, 27.9%), learning disability (n = 70, 21.2%), anxiety (n = 53, 16.1%), autism spectrum disorder (n = 35, 10.6%), and tic (n = 18, 5.5%).

A total of 270 (81.8%) patients concomitantly used other pharmacological treatments for ADHD and comorbid psychiatric conditions while on GXR. The most frequently used medications included methylphenidate (n = 123, 37.3%), lisdexamfetamine (n = 121, 36.7%), melatonin (n = 26, 7.9%), centrally acting sympathomimetics (n = 24, 7.3%), apriprazole (n = 25, 7.6%), and risperidone (n = 18, 5.5%). Six (1.8%) patients used clonidine, 1 (0.3%) patient used trazodone, and 40 patients used SSRIs (citalopram, n = 3, 0.9%; escitalopram, n = 13, 13.9%; fluoxetine, n = 16, 4.8%; paroxetine, n = 1, 0.3%; sertraline, n = 7, 2.1%).

### Characteristics of GXR treatment in the study

The most common reasons for GXR initiation were non-optimal control of ADHD symptoms, to improve response to stimulants, to extend duration of effect of stimulants, and to avoid increasing doses of ADHD stimulants (Additional file 1: Table S2). Compared with 60 (18.2%) patients receiving monotherapy, 270 (81.8%) received GXR adjunctive therapy. The median daily dose of GXR at initiation was 1.0 mg. Of the 325/330 (98.5%) patients who changed GXR dose during post-GXR initiation observation, 307/325 (94.5%) had an increase and 18/325 (5.5%) had a decrease. The mean (SD) total dose change for all patients was 2.4 mg (0.5 mg). The mean (SD) length of GXR treatment was 7.9 (3.6) months and was similar between children (n = 242) and adolescents (n = 88). After a median (range) of 8.4 (0.2–12) months, almost 80% of patients were continuing GXR treatment (~ 20% discontinued). The most common reasons for discontinuation were safety/tolerability issues.

### Primary analysis: assessments of symptoms and functionality while receiving GXR

**Fig. 1:**
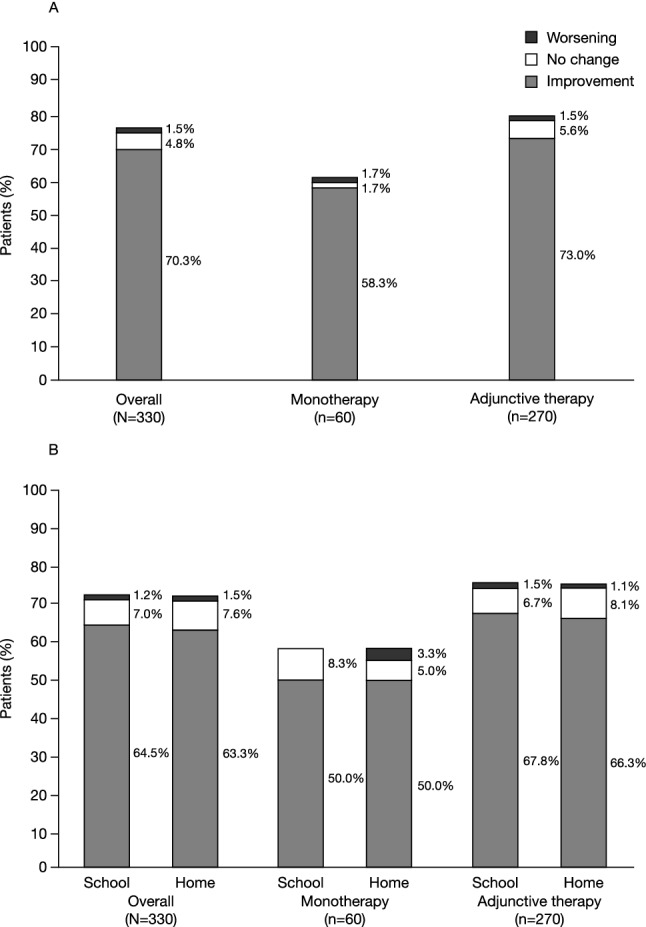
**A** ADHD symptoms and **B** functionality while receiving GXR: overall and by therapy type (monotherapy or adjunctive therapy). Symptoms and functionality assessed in the overall population was the primary analysis; assessment by therapy type was a post-hoc analysis. Best symptom response while on treatment with GXR was reported. Data for ADHD symptom assessment were derived from ADHD rating scales for 313 patients and from clinical notes for 17 patients. Data for ADHD functional assessment were derived from ADHD rating scales for 309 patients and from clinical notes for 21 patients. Adjunctive therapy was defined as patients receiving concomitant pharmacologic ADHD treatments. Percentages are based on the total number of enrolled patients. Due to missing values (≥ 6 months’ follow-up was required for inclusion in the study; patients may have discontinued between months 6 and 12), percentages in each category do not add up to 100%. *ADHD* attention-deficit/hyperactivity disorder, *GXR* guanfacine extended-release

Improvements in clinical outcomes were reported for > 70% of patients receiving GXR (Fig. 1). No change/worsening of ADHD symptoms was observed for < 5% of patients receiving GXR (missing data, 77 [23.3%]). Additionally, > 60% of patients showed improvements in school performance and homelife while no change/worsening in school performance and homelife functionality was reported for < 10% of patients.

### Safety and tolerability

Overall, 349 TEAEs were experienced by 147/330 (44.5%) patients (Table 1). The majority were non-serious (341/349, 97.7%), mild in severity (n = 314, 90.0%), and related to GXR treatment (n = 263, 75.4%). No action was deemed necessary regarding management of GXR treatment for 53% of TEAEs. The most common TEAEs (reported in ≥ 5% of patients) were somnolence (n = 59/330, 17.9%), headache (n = 34/330, 10.3%), insomnia (n = 26/330, 7.9%), presyncope (n = 22/330, 6.7%), and decreased appetite (n = 18/330, 5.5%). Eight serious TEAEs were experienced by 7 patients (Table 1). Of these, 6/8 events were moderate in severity, 7/8 were related to GXR treatment, and 7/8 were reported as resolved. A total of 4 serious TEAEs experienced by 3 patients led to GXR discontinuation, 1 serious TEAE resulted in dose interruption, and 1 serious TEAE resulted in dose reduction. No action was deemed necessary regarding GXR management for 2 serious TEAEs experienced by 2 patients.

**Table 1:** Summary of incidence of TEAEs and serious TEAEs

Parameter	Total (N = 330)		
	N of events	N of patients	% patients
Total TEAEs	349	147	44.5
Seriousness			
Non-serious	341	145	43.9
Serious^a^	8	7	2.1
Severity			
Mild	314	132	40.0
Moderate	29	23	7.0
Severe	6	5	1.5
Relationship to study drug			
Related	263	126	38.2
Not related	86	55	16.7
Action taken			
No action taken	185	92	27.9
Dose reduced	60	37	11.2
Dose increased	50	33	10.0
Dose interrupted	6	4	1.2
Treatment discontinued	48	32	9.7
Outcome			
Resolved	299	133	40.3
Resolved with sequelae	0	0	0.0
Resolving	19	16	4.8
Not resolved	22	18	5.5
Death	0	0	0.0
Unknown	9	9	2.7

*TEAE* treatment-emergent adverse event

^a^Serious TEAEs were hypotension (2 events), suicidal ideation (2 events), sinus bradycardia (1 event), aggression (1 event), agitation (1 event), and insomnia (1 event)

### Post*-*hoc analyses: assessments of symptoms and functionality in patient subgroups

Improvements in ADHD symptoms were observed in 35/60 (58.3%) patients receiving monotherapy and 197/270 (73.0%) patients receiving GXR adjunctive therapy. Similar improvements were seen in functionality assessments of school performance (monotherapy, 30/60 [50.0%]; adjunctive therapy, 183/270 [67.8%]) and homelife (monotherapy, 30/60 [50.0%]; adjunctive therapy, 179/270 [66.3%]).

**Fig. 2:**
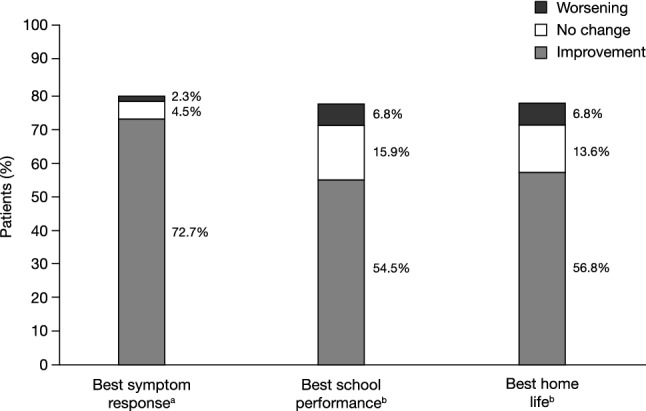
Best ADHD symptom and functional response (post-hoc subgroup analyses) (n = 44). Post-hoc analyses examined best symptom response and best functional response while on treatment with GXR among patients who were initiated on GXR to reduce the use of atypical antipsychotics. Percentages were based on the total number of overall patients. Due to missing values (≥ 6 months’ follow-up was required for inclusion in the study; patients may have discontinued between months 6 and 12), percentages in each category do not add up to 100%. ^a^Based on General Physician ADHD Symptom Assessment. ^b^Based on General Physician Functional Assessment. *ADHD* attention-deficit/hyperactivity disorder, *GXR* guanfacine extended-release

Of the 44 patients initiated on GXR to reduce use of AAPs, ADHD symptom improvements were experienced by > 70%, including > 55% of patients in school performance and homelife functionality (Fig. 2). AAPs used included risperidone, quetiapine, and aripiprazole.

Patients with comorbid autism spectrum disorder, oppositional defiant disorder, anxiety, or learning disability experienced improvements in ADHD symptoms and functionality consistent with the primary findings (Table 2).

**Table 2:** Best symptom and functionality response while receiving GXR, by psychiatric comorbidities of interest (post-hoc analyses)

Comorbidity (MedDRA preferred term)	Improvement, n (%)^a^		
	ADHD symptom assessment^b^	Functionality assessment^b^	
		School performance	Homelife
Oppositional defiant disorder, n = 92	64 (69.6)	54 (58.7)	51 (55.4)
Learning disability, n = 70	52 (74.3)	51 (72.9)	48 (68.6)
Anxiety, n = 53	39 (73.6)	38 (71.7)	36 (67.9)
Autism spectrum disorder, n = 35	24 (68.6)	24 (68.6)	21 (60.0)

*ADHD* attention-deficit/hyperactivity disorder, *GXR* guanfacine extended-release, *MedDRA* Medical Dictionary for Regulatory Activities

^a^Percentages were calculated using the number of patients in the respective comorbidity categories as denominator

^b^General physician assessment

### Exploratory analyses: assessments in symptoms and functionality in patients who had received prior ADHD pharmacotherapy

In the subgroup who had received prior pharmacotherapy, a greater proportion of patients who had reported worsening ADHD symptoms on prior pharmacotherapy experienced improvements on GXR compared with those reporting no changes (Table 3). Similarly, a greater proportion of patients reporting worsening changes in homelife functionality and school performance on prior pharmacotherapy experienced improvements on GXR compared with those reporting no changes.

**Table 3:** Categorization of clinical outcomes (ADHD symptoms and functionality)—exploratory subgroup analysis (n = 304)

Pre-initiation of GXR treatment^a^		Post-initiation of GXR treatment^b^		
	n	Improvement	No change	Worsening
General Physician Symptom Assessment, n (%)*^c^				
Improvement	172	130 (75.6)	2 (1.2)	0 (0.0)
No change	74	37 (50.0)	9 (12.2)	1 (1.4)
Worsening	54	44 (81.5)	2 (3.7)	3 (5.6)
General Physician Functional Assessment, n (%)^c^				
Homelife*				
Improvement	132	102 (77.3)	4 (3.0)	1 (0.8)
No change	118	52 (44.1)	9 (7.6)	2 (1.7)
Worsening	48	34 (70.8)	9 (18.8)	1 (2.1)
School performance*				
Improvement	161	111 (68.9)	3 (1.9)	0 (0.0)
No change	89	45 (50.6)	11 (12.4)	2 (2.2)
Worsening	44	33 (75.0)	5 (11.4)	2 (4.5)

Exploratory subgroup analysis in a subgroup of patients who had received prior pharmacotherapy for ADHD

*ADHD* attention-deficit/hyperactivity disorder, *GXR* guanfacine extended-release

^*^P < 0.001, derived through extension of McNemar’s test (Bowker’s test)

^a^Overall response to treatment regimen administered prior to GXR administration

^b^The best response registered while on treatment with GXR

^c^Percentages were calculated using the corresponding categories under GXR treatment count as denominator; due to missing values, percentages in each category do not add up to 100%

## Discussion

This retrospective chart review deepens our understanding of GXR use beyond the clinical trial setting, suggesting that children and adolescents with ADHD receiving GXR in real-world clinical practice experienced improvements in ADHD symptoms and functionality. These improvements were evident for the overall population (primary analysis) and patients who had experienced worsening symptoms or functionality with ADHD pharmacotherapy immediately before GXR initiation (exploratory analysis). Our data suggest that, in Canada, GXR is less commonly used as monotherapy than as adjunctive treatment. Post-hoc analyses demonstrated improvements in ADHD symptoms in 58.3% of patients using GXR as monotherapy and 73.0% of patients using GXR as adjunctive therapy, corroborating randomized, double-blind, placebo-controlled, clinical trial data demonstrating that GXR is clinically efficacious as either monotherapy or adjunctive therapy [18–25].

GXR is a selective α_2_-adrenoceptor agonist. The majority of α_2_-adrenoceptors are expressed at postsynaptic neurons of the prefrontal cortex, and have been shown to regulate cognitive functions [32]. Although the exact mechanism of action of GXR is unknown, it is thought that GXR stimulation of these receptors may have an effect on ADHD symptoms related to working memory, attention regulation, and response inhibition [33, 34]. This mechanism may explain its efficacy as monotherapy in patients who cannot tolerate or do not respond to stimulants and also when used with stimulants as adjunctive therapy [14, 23]. Stimulant treatment is considered the mainstay of ADHD therapy; however, approximately 25–30% of patients may not adequately respond to stimulants [11, 12]. Stimulants used to treat ADHD (e.g., amphetamine and methylphenidate) act via different mechanisms than GXR, inhibiting the reuptake of dopamine and norepinephrine and/or enhancing their release [33].

In this study, a relatively high proportion of patients continued treatment beyond 12 months (263/330, 79.7%). For comparison, albeit from different populations and methodologies, three large database analyses from Taiwan, Canada, and the United Kingdom found persistence with stimulant and non-stimulant ADHD medications at 1 year ranged from 41.2 to 77.0% [35–37]. We acknowledge that in our study, continuing treatment was assessed by prescription receipt rather than by refill. However, the rewriting of prescriptions could suggest satisfaction with the effects of GXR, a theory supported by our primary analysis.

The observed safety profile in our study is generally consistent with that described in the GXR product monograph (Intuniv XR^®^) [15], including rates of somnolence, headache, insomnia, and decreased appetite. However, rates of hypotension (1.8%) and orthostatic hypotension (0.6%) in this study were generally lower than those reported in the product monograph for patients who received GXR in clinical trials (0.7–2.5% and 1.0–3.8%, respectively). This difference may be attributed to our data originating in a real-world setting in which adverse event reporting was collected from 10 heterogeneous sites, as opposed to being prospectively reviewed in a clinical trial setting. Other potential explanations include more patients receiving GXR as adjunctive treatment to stimulants in our study (as stimulants are associated with small increases in blood pressure) [14], or that up-titration was conducted slowly and at low increments. Timings of TEAEs during the course of therapy were not provided, which leaves open the possibility that some effects, such as somnolence, may have occurred at treatment initiation and then resolved.

Many physicians in Canada use AAPs in complicated or comorbid cases of ADHD [4]; however, there are concerns about metabolic side effects (requiring lab-based monitoring) associated with AAPs [38, 39], and data are lacking regarding the efficacy of AAPs in treating ADHD symptoms [4]. In this study, 44 patients were initiated on GXR by their physicians in order to lower the doses of AAPs, with post-hoc analyses showing that > 70% of these patients experienced ADHD symptom improvements. These findings may therefore be useful for informing future treatment decisions for patients with ADHD.

This was a real-world study of medical chart review data from patients with ADHD, and so included patients from a naturalistic setting. This means that, unlike in the clinical trial setting where strict exclusion/inclusion criteria are applied, patients who would be typically seen in clinical practice were included, such as those with confounding comorbidities. Here, many patients were reported to have ≥ 1 comorbid psychiatric condition, including oppositional defiant disorder (27.9%), learning disability (21.2%), anxiety (16.1%), and autism spectrum disorder (10.6%). The results of our post-hoc analysis suggest that GXR is effective in improving ADHD symptoms and functionality in patients with ADHD with different psychiatric comorbidities or without comorbid psychiatric conditions. These findings are consistent with another study showing GXR to be effective in reducing ADHD symptoms in patients with ADHD and comorbid oppositional symptoms [21], and warrant further investigation.

The limitations of the study reported here include the inherent limitations of retrospective medical record analysis; the patients selected for study inclusion represented a convenience sample. The vast majority of patients were from Ontario and 82% of participants were Caucasian. Findings may therefore not be generalizable to or fully representative of the broader ADHD population in Canada. The analysis of symptom improvement during previous therapy was exploratory and limited to improvement, no change, or worsening from the overall response to the last treatment regimen. In addition, as this was a real-world study, outcomes may have been affected by factors such as adherence, access, long-term duration of therapy, and the presence of comorbidities and concomitant medications. However, compared with patients included in clinical trials, this population may represent a more accurate view of typical patients seen in routine practice.

Although data from different sites were standardized by transcription onto CRFs, it should be acknowledged that there may be inherent differences in data collection methods. Specifically, some sites used standardized methodology such as rating scales that could be used to support and analyze data further, but these data were not available for all patients or sites. Also, although the data were interpreted as accurately as possible using available clinical notes, some assumptions were made. Therefore, study findings should be interpreted in consideration of these limitations.

The maximum dose of GXR is 7 mg daily for adolescents when used as monotherapy. However, in our study, the highest dose reported was only 5 mg. This observation may relate to the fact that GXR was predominantly used as adjunctive therapy (for which the maximum recommended dose is 4 mg), or perhaps the clinicians treating these patients adopted a conservative approach to GXR dosing.

## Conclusions

In routine clinical practice in Canada, most children and adolescents with ADHD treated with GXR experienced improvements in ADHD symptoms, and in functionality at both school and home. Safety data in this chart review are consistent with those reported in clinical trials. Additionally, this real-world evidence corroborates the efficacy of GXR observed in randomized controlled trials and demonstrates the effectiveness of GXR as adjunctive therapy and, albeit with a smaller sample size, as monotherapy.

## Supplementary Information

**Additional file 1: Table S1.** Patient disposition and sociodemographics. **Table S2.** Characteristics of GXR treatment from initiation through end of study.

## Data Availability

The data that support the findings of this study are available from JSS Medical Research Inc., but restrictions apply to the availability of these data, which were used under license for the current study, and so are not publicly available. Data are however available from the authors upon reasonable request and with permission of Takeda Canada Inc.

## Abbreviations

AAP: Atypical antipsychotics
ADHD: Attention-deficit/hyperactivity disorder
CI: Confidence interval
Conners 3-P: Conners 3-Parents
CRF: Case report form
GXR: Guanfacine extended-release
MedDRA: Medical Dictionary for Regulatory Activities
NICHQ: National Institute for Children’s Health Quality
SD: Standard deviation
SNAP-IV-26: Swanson, Nolan, and Pelham-IV
TEAE: Treatment-emergent adverse event
WFIRS-P: Weiss Functional Impairment Rating Scale-Parent Report
