# Supplementary material for: A retrospective medical chart review of clinical outcomes in children and adolescents with attention-deficit/hyperactivity disorder treated with guanfacine extended-release in routine Canadian clinical practice
Source: Child Adolesc Psychiatry Ment Health. 2021 Oct 4;15:55. doi: 10.1186/s13034-021-00402-5 (PMC8491395; doi:10.1186/s13034-021-00402-5)
Supplement: Supplementary file 1 — Additional file 1: Table S1. Patient disposition and sociodemographics. Table S2. Characteristics of GXR treatment from initiation through end of study. [file 13034_2021_402_MOESM1_ESM.docx]

# Additional file 1

### Table S1 Patient disposition and sociodemographics

| Disposition | Total (N=330) |
| --- | --- |
| Screened, n (%) | 330 (100.0) |
| Enrolled, n (%) | 330 (100.0) |
| Completed the study, n (%)^a^ | 263 (79.7) |
| Pre-initiation of GXR treatment, n (%)  Had prior pharmacologic treatment  Had prior non-pharmacologic treatment | 308 (93.3)  304 (92.1)  133 (40.3) |
| Parameter |  |
| Age (years)  Available, n  Mean (SD)  Median (range)  95% CI of mean | 330  10.9 (2.63)  10.7 (5.7^b^–17.9)  (10.58–11.15) |
| Age categories,^c^ n (%) |  |
| Children  Adolescents | 242 (73.3)  88 (26.7) |
| Sex, n (%) |  |
| Female  Male | 78 (23.6)  252 (76.4) |
| Race, n (%) |  |
| Caucasian  African American  Asian  Hispanic  Other  Unknown | 269 (81.5)  7 (2.1)  8 (2.4)  1 (0.3)  15 (4.5)  30 (9.1) |
| Province, n (%) |  |
| ON  QC  NS | 322 (97.6)  7 (2.1)  1 (0.3) |
| Location of residence, n (%) |  |
| Urban  Rural | 282 (85.5)  48 (14.5) |

^a^For inclusion in the study, ≥6 months’ follow-up was required; patients may have discontinued between months 6 and 12

^b^One patient aged 5.7 years was included in the analysis

^c^Children (aged 6–12 years); adolescents (aged 13–17 years)

CI, confidence interval; NS, Nova Scotia; ON, Ontario; QC, Quebec; SD, standard deviation

### Table S2 Characteristics of GXR treatment from initiation through end of study

| Parameter | Children  (n=242) | Adolescents  (n=88) | Total  (N=330) |
| --- | --- | --- | --- |
| Dose of GXR at treatment initiation, mg |  |  |  |
| Available, n  Mean (SD)  Median (range)  95% CI of mean | 242  1.1 (0.43)  1.0 (1.0–4.0)  1.05–1.16 | 88  1.1 (0.53)  1.0 (1.0–5.0)  0.97–1.19 | 330  1.1 (0.46)  1.0 (1.0–5.0)  1.05–1.15 |
| Reasons for GXR treatment initiation, n (%)^a^ |  |  |  |
| Non-optimal control  Reduce atypical antipsychotics  Appearance of psychiatric comorbidities  Avoid increasing doses of stimulants  Improve response to stimulants  Extend duration  Other | 215 (88.8)  33 (13.6)  17 (7.0)  45 (18.6)  94 (38.8)  79 (32.6)  31 (12.8) | 79 (89.8)  11 (12.5)  9 (10.2)  24 (27.3)  42 (47.7)  33 (37.5)  10 (11.4) | 294 (89.1)  44 (13.3)  26 (7.9)  69 (20.9)  136 (41.2)  112 (33.9)  41 (12.4) |
| Patients with GXR changes, n (%) | 240 (99.2) | 85 (96.6) | 325 (98.5) |
| Change in dose of GXR b  Available, n  Mean (SD)  95% CI of mean  Change in frequency of GXR, n (%), QD^b^ | 240  2.3 (0.53)  2.25–2.39  240 (99.2) | 85  2.6 (0.52)  2.49–2.71  85 (96.6) | 325  2.4 (0.54)  2.33–2.45  325 (98.5) |
| Status of GXR treatment, n (%)b |  |  |  |
| Ongoing  Discontinued | 189 (78.1)  53 (21.9) | 74 (84.1)  14 (15.9) | 263 (79.7)  67 (20.3) |
| Reason for discontinuation, n (%)^c^ |  |  |  |
| Safety/tolerability issues  Treatment failure/ineffectiveness  Reimbursement  Patient/parent decision  Loss to follow-up  Other | 13 (5.4)  13 (5.4)  2 (0.8)  6 (2.5)  2 (0.8)  17 (7.0) | 6 (6.8)  1 (1.1)  0 (0.0)  3 (3.4)  0 (0.0)  4 (4.5) | 19 (5.8)  14 (4.2)  2 (0.6)  9 (2.7)  2 (0.6)  21 (6.4) |
| Length of GXR exposure (months) |  |  |  |
| Available, n  Mean (SD)  Median (range)  95% CI of mean | 242  7.9 (3.61)  8.4 (0.2–12)  7.49–8.41 | 88  7.7 (3.53)  8.4 (0.5–12)  6.97–8.47 | 330  7.9 (3.58)  8.4 (0.2–12)  7.50–8.28 |

^a^More than one reason for GXR initiation may have been reported

^b^More than one change in dose, frequency, status, or discontinuation reason may have been reported (≥6 months’ follow-up was required for inclusion in the study; patients may have discontinued between months 6 and 12)

^c^The last available reasons for discontinuation were considered for patients who discontinued treatment. Percentages were based on n/N values in each column header

CI, confidence interval; GXR, guanfacine extended release; QD, once daily; SD, standard deviation
